# Multimodal interaction enhanced representation learning for video emotion recognition

**DOI:** 10.3389/fnins.2022.1086380

**Published:** 2022-12-19

**Authors:** Xiaohan Xia, Yong Zhao, Dongmei Jiang

**Affiliations:** ^1^Shaanxi Key Laboratory on Speech and Image Information Processing, National Engineering Laboratory for Integrated Aero-Space-Ground-Ocean Big Data Application Technology, School of Computer Science, Northwestern Polytechnical University, Xi'an, Shaanxi, China; ^2^Pengcheng Laboratory, Shenzhen, Guangdong, China

**Keywords:** emotion recognition, representation learning, cross-modal interaction, cross-attention, semantic enhancement

## Abstract

Video emotion recognition aims to infer human emotional states from the audio, visual, and text modalities. Previous approaches are centered around designing sophisticated fusion mechanisms, but usually ignore the fact that text contains global semantic information, while speech and face video show more fine-grained temporal dynamics of emotion. From the perspective of cognitive sciences, the process of emotion expression, either through facial expression or speech, is implicitly regulated by high-level semantics. Inspired by this fact, we propose a multimodal interaction enhanced representation learning framework for emotion recognition from face video, where a semantic enhancement module is first designed to guide the audio/visual encoder using the semantic information from text, then the multimodal bottleneck Transformer is adopted to further reinforce the audio and visual representations by modeling the cross-modal dynamic interactions between the two feature sequences. Experimental results on two benchmark emotion databases indicate the superiority of our proposed method. With the semantic enhanced audio and visual features, it outperforms the state-of-the-art models which fuse the features or decisions from the audio, visual and text modalities.

## 1. Introduction

Automatic emotion recognition, as the first step to enable machines to have emotional intelligence, has been an active research area for the past two decades. Video emotion recognition (VER) refers to predicting the emotional states of the target person by analyzing information from different cues such as facial actions, acoustic characteristics and spoken language (Rouast et al., 2019; Wang et al., 2022). At the heart of this task is how to effectively learn emotional salient representations from multiple modalities including audio, visual, and text.

Previous works usually extract modality-specific features, such as the word-level embeddings from text (Pennington et al., 2014), and frame-level acoustic features from speech (Degottex et al., 2014) or appearance descriptors from face images (Baltrusaitis et al., 2018), then use various fusion strategies to explore the temporal dependencies among the feature sequences of different modalities. For instance, the bidirectional cross-attention proposed by Tsai et al. (2019) to attend interactions between any two pair-wise feature sequences, was extended by Zheng et al. (2022) to implement interactions between three modalities by connecting the cross-attention modules in series. In He et al. (2021), the time squeeze fusion was proposed to model the time-dependent modality-specific interactions. In these works (Tsai et al., 2019; He et al., 2021; Zheng et al., 2022), the audio, visual, and text modalities were treated as three time-series that play the same role. Several works proposed to first fuse the audio and visual feature sequences into a higher level space, then fuse this bimodal feature sequence with the textual feature sequence (Fu et al., 2022; Zhang et al., 2022). Alternatively, text-centered frameworks were designed to explore the cross-modal interactions between textual and non-textual feature sequences (Han et al., 2021; He and Hu, 2021; Wu et al., 2021). In the works above, the textual features are feature sequences composed of the word-level embeddings. In fact, the whole sentence contains more accurate semantics than the word-level embeddings. Accordingly, the challenge is how to effectively leverage textual emotion information while preserving the high-level global semantics. Facing this challenge, Sun et al. (2020) adopted the pre-trained BERT model (Devlin et al., 2019) to obtain global text embeddings and two long-short term memory (LSTM) models to extract sentence-level audio and visual features independently, then modeled the correlations between the outer-product matrices of text-audio and text-visual features to learn the multimodal representations. In Dai et al. (2020), three LSTMs were used to get the global representations of audio, visual, and text modality, respectively. Meanwhile, a set of emotion embeddings was constructed for each modality, representing the semantic meanings for the emotion categories to be recognized. Specifically, the pre-trained GloVe (Pennington et al., 2014) embeddings of emotion category words (happy, sad, etc) were used as textual emotion embeddings, which were mapped to obtain the audio and visual emotion embeddings, respectively, through two learnable mapping functions. Then, the similarity score between the emotion embeddings and the global representation was calculated for each modality separately, and finally fused to get the emotion prediction. This work leveraged the global semantic information, however, the semantics contained in the emotion category words are less goal-oriented toward the target emotion and the important cross-modal feature interactions are ignored.

In fact, as a complex psychological and physiological phenomenon, emotion can be pre- and post-cognitive: initial emotional responses produce thoughts, which produce affect (Lerner and Keltner, 2000). From this perspective, the process of emotional expression, either through facial expression or the way of speaking, is implicitly regulated by the semantic information. Therefore, in this work, we propose a semantically enhanced module for audio or visual encoders, striving to learn more emotion-relevant features from individual video frames or speech segments with the guidance of high-level semantic information from text.

Additionally, in order to capture the temporal dynamics in audio and video signals, sequential learning is usually performed over the unimodal or concatenated features (Dai et al., 2021; Nguyen et al., 2021). However, such approach lacks information exchanging between the audio and visual sequential features. A classical solution is based on the bidirectional cross-attention between the pair-wise modalities (Tsai et al., 2019). Nevertheless, the redundancy that exists in audio and video signals is ignored, moreover, the bidirectional cross-attention leads to additional computational complexity. In the field of video understanding, the Multimodal Bottleneck Transformer (Nagrani et al., 2021; Liu et al., 2022) was recently proposed for audiovisual fusion with the advantage of condensing relevant unimodal information and meanwhile reducing the computational cost. Inspired by this, we adopt the bottleneck Transformer to reinforce the audio and visual features, by leveraging attention bottlenecks as a bridge to explore the temporal interactions between the two modalities. By doing so, our model can simultaneously consider exchanging complementary information and reducing redundancy during the coordinate representation learning process of audio and visual modalities.

Overall, we propose a representation learning approach for video emotion recognition that achieves dual-enhancement through multimodal interactions. First, the encoders of audio and visual modalities are enhanced by the global semantic information in text. Then, the audio and visual feature sequences are reinforced again with the complementary information of each other. Finally, the attentive decision fusion is performed to obtain the final emotion prediction. The effectiveness of the proposed method is verified by extensive experiments on two widely used emotion datasets, i.e., IEMOCAP (Busso et al., 2008) and CMU-MOSEI (Zadeh and Pu, 2018). In summary, the contributions are summarized as follows:

- We propose a semantic enhancement module for the audio and visual feature encoder to enhance the audio and visual features under the guidance of global semantics from the text modality. The enhanced audio and visual features contain more emotion-relevant information.
- To achieve efficient cross-modal interaction between temporal audio and visual feature sequences, the bottleneck Transformer is adopted as the cross-modal encoder. Specifically, the bottleneck Transformer reinforces audio and visual representations by modeling their dynamic interactions and meanwhile reducing redundancy in the temporal sequences.
- We conduct extensive experiments on two benchmarks and the results demonstrate the superiority of our proposed method for video emotion recognition.

The remainder of this paper is organized as follows. Section 2 reviews the previous related works on video emotion recognition. Section 3 explains our proposed framework in detail. Section 4 reports the experiment results, followed by the conclusions and future work in Section 5.

## 2. Related works

### 2.1. Feature representations for video emotion recognition

Extracting effective feature representations is the first and foremost step in video emotion recognition. By considering the heterogeneity of different modalities in the video, separate models are used to extract unimodal features from the raw data of each modality. For text modality, with the advances in natural language processing, pre-trained models such as Word2Vec (Mikolov et al., 2013) and BERT (Devlin et al., 2019) are commonly used for word embedding. As for audio and visual modalities, various hand-crafted features have been designed based on corresponding domain knowledge, such as acoustic descriptors including prosodic and spectral related parameters (Degottex et al., 2014) and visual features based on facial landmarks, facial action units, etc. (Baltrusaitis et al., 2018). Alternatively, benefiting from the development of deep learning, deep-learned feature representations based on the large-scale pre-trained convolutional neural networks (CNN) such as ResNet (He et al., 2016) and VGGish (Hershey et al., 2017) also have been widely used for emotion recognition (Alisamir and Ringeval, 2021; Li and Deng, 2022). Compared with those hand-crafted features, the pre-trained CNN encoders can extract more powerful visual/audio features. However, the general encoding of versatile CNNs does not consider the speciality of emotion and may further limit the emotional representation ability of extracted deep features.

Recently, Nguyen et al. (2021) proposed a two-stream auto-encoder architecture to learn compact yet representative features from audio and visual raw data individually. Then the learned audio and visual features are concatenated and fed into an LSTM for sequential learning and predicting the dimensional emotion scores. In Hazarika et al. (2020), shared-private representations were learned through two separate encoders by projecting each modality to modality-invariant and -specific subspaces, then a Transformer was used to fuse these features into a joint vector for final prediction. By decoupling the common and specific patterns in audio, visual, and text modalities, the learned shared-private representations were highly effective in reducing the modality gap and contributed to significant gains. Self-supervised representation learning also has been adopted for emotion recognition. For instance, Yu et al. (2021) leveraged self-supervised multi-task learning strategy to learn modality-specific representations. Through joint training the multimodal and uni-modal tasks, this model learned the consistency and difference between different modalities simultaneously.

Our work aims at representation learning enhanced with multimodal interactions. Different from previous work, we leverage the high-level global semantics extracted from text modality to guide the representation learning of audio and visual encoders, and therefore the learned audio/visual features could contain more emotion-related information.

### 2.2. Multimodal fusion for video emotion recognition

Multimodal fusion is another core challenge for video emotion recognition. Early works usually adopted the traditional feature-level or decision-level fusion methods (Ma et al., 2019; Zhang et al., 2019, 2021b; Sharma and Dhall, 2021). With the rise of attention mechanisms, recent works are mostly focusing on cross-modal interactions to explore more effective fusion strategies.

In Tsai et al. (2019), the powerful Transformer network was introduced to multimodal emotion recognition task, to take its advantage of modeling long-term dependencies across modalities. The authors adopted the Transformer decoder-like module to fuse cross-modal information between any two paired modalities by latently adapting one modality to another. To further mine the cross-modal interactions between two or three modalities simultaneously, Zheng et al. (2022) proposed cascade multi-head attention for full fusion of multimodal features by connecting attention modules in series and regarding different modality features as query for different attention modules.

The above-mentioned works focus on exploring the interactions between different modalities by treating audio, visual, and text modalities equally. Another type of representative works argues that text plays a more important role than audio and visual modalities and designs diverse text-centered frameworks for multimodal emotion recognition. In Han et al. (2021), the authors proposed a Transformer-based bi-bimodal fusion network, consisting of two text-related complementing modules, to separately fuse textual feature sequence with audio and visual feature sequences. In Wu et al. (2021), two cross-modal prediction modules, i.e., text-to-visual and text-to-audio models, were designed to decouple the shared and private information of non-textual modalities compared to the textual modality. The shared non-textual information was used to enrich the semantics of textual features and the private non-textual features were later fused with the enhanced textual features through a regression layer for final prediction.

Apart from regarding text as the central modality that plays the most important role among the three modalities, several researchers take into account the difference between audio-visual and text modalities in terms of information granularity. For instance, Fu et al. (2022) proposed a non-homogeneous fusion network by first fusing audio and visual feature sequences through an attention aggregation module and then fusing audio-visual features with textual feature sequence *via* cross-modal attention. Similarly, Zhang et al. (2022) proposed a hierarchical cross-modal encoder module to gradually fuse the modality features. Specifically, an adversarial multimodal refinement module was designed to decompose each modality-specific features to common and private representations. The audio and visual private features were first fused, then this joint audio-visual feature sequence was fused with the textual feature sequence, and finally the fused private features were fused with the common features, resulting in the final joint multimodal representation.

Different from these related works, we are inspired by the emotion expression process that both facial expressions and intonations are implicitly regulated by high-level semantics, and propose a semantic enhancement module to leverage the textual high-level semantics to guide audio and visual representations. In addition, these semantically enhanced audio and visual representations are further reinforced through a multimodal bottleneck Transformer module to exchange their complementary information while reducing redundancy.

## 3. Proposed method

**Figure 1:**
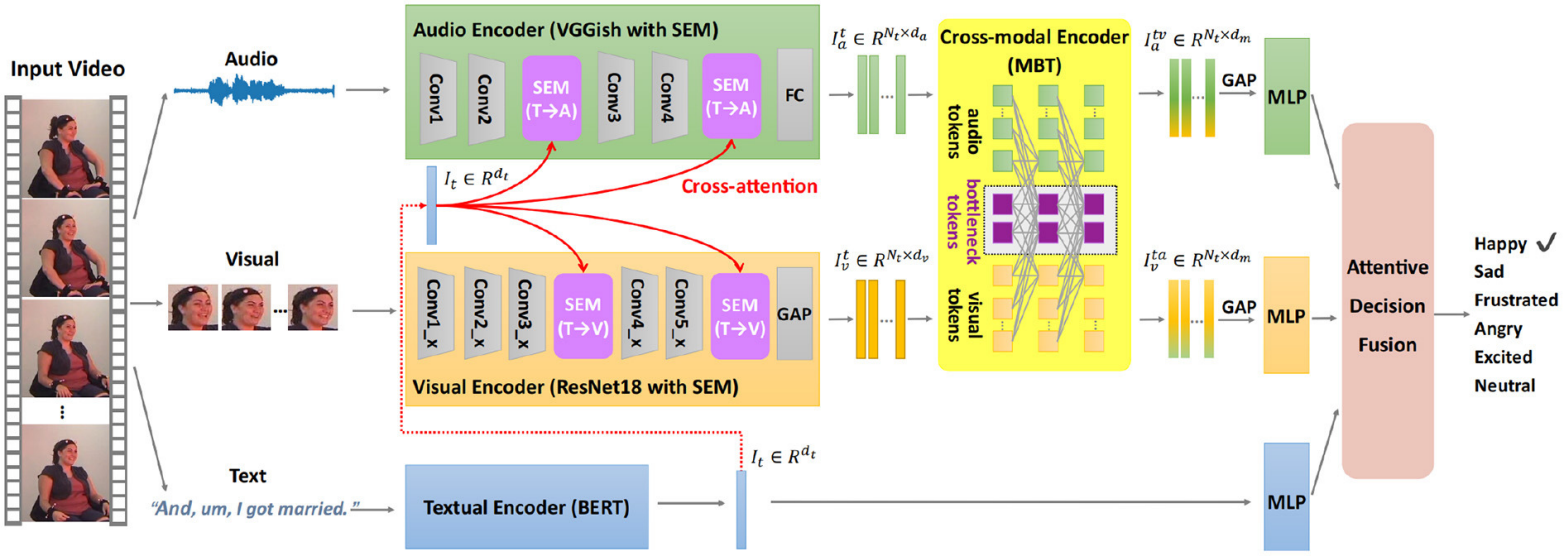
The proposed end-to-end multimodal emotion recognition (MER) framework with the semantic enhancement module (SEM) and multimodal bottleneck Transformer (MBT), is denoted as MER-SEM-MBT. Given a facial video clip, the global semantic feature is first extracted through the textual encoder, which is used to guide the audio and visual representation learning through the semantic enhancement module. Then the cross-modal encoder is adopted to reinforce audio and visual representations through temporal cross-modal interaction *via* a multimodal bottleneck Transformer. Lastly, three separate multi-layer perceptrons (MLPs) are implemented to get unimodal decisions from audio, visual, and text modalities, respectively. Attentive fusion is performed to aggregate these decisions for final emotion prediction. The example facial video is from IEMOCAP dataset (Busso et al., 2008).

Figure 1 depicts the architecture of the proposed multimodal emotion recognition (MER) framework with the semantic enhancement module (SEM) and multimodal bottleneck Transformer (MBT), denoted as MER-SEM-MBT. Specifically, we first extract global textual features *via* the textual encoder to represent the high-level semantics, which is used in the SEM to guide the audio/visual encoder to learn emotionally relevant audio/visual features. These semantically enhanced audio and visual feature sequences are sent into the cross-modal encoder to mutually reinforce their representations through cross-modal interaction *via* a bottleneck Transformer. The reinforced audio and visual features are then separately input into a global average pooling (GAP) layer which is followed by a multi-layer perceptron (MLP) to output unimodal decisions. In the meanwhile, the global textual features are fed into another MLP to get the textual decision. Finally, attention-based decision fusion is adopted for the final emotion prediction.

The details are explained in the following subsections.

### 3.1. Unimodal encoder

For emotion recognition from text, one must analyze the affective state from the complete sentence rather than individual words or phrases. In contrast, regarding the audio and visual modalities, a single video frame or a speech segment longer than 250 ms (Provost, 2013) may contain meaningful emotion information. Therefore, when designing the unimodal encoders, the global semantic features are extracted from the transcripts of the sentences, the audio feature sequence is extracted from the temporal segments, and the visual feature sequence is extracted at the frame level.

#### 3.1.1. Textual encoder

With the advent of Transformer, pre-trained large models such as BERT provided a new paradigm for dynamic text feature encoding based on contextual information with the help of the self-attention mechanism. Therefore, we use the pre-trained BERT model provided in the HuggingFace library (Wolf et al., 2020) as textual encoder. Specifically, the class token (“CLS”) of the output layer is adopted as the high-level semantic features $I_{t}\in {\mathbb{R}}^{d_{t}}$, where *d*_*t*_ = 768.

#### 3.1.2. Audio encoder

We first calculate the log mel-spectrogram by utilizing 64 Mel filters on the spectrum obtained from the Short-Time Fourier Transform, with a window size of 25 ms and a hop of 10 ms. Then the log mel-spectrogram is split into segments of 960 ms, each of which is fed into the pre-trained VGGish (Hershey et al., 2017) network, outputting a 128-dimensional feature vector from the last fully-connected layer. Therefore, for an audio clip of *l* s, the audio feature sequence $I_{a}\in {\mathbb{R}}^{N_{t}\times d_{a}}$ is obtained, with the sequence length *N*_*t*_ = *l*/0.96 and *d*_*a*_ = 128.

#### 3.1.3. Visual encoder

The input of visual encoder is a facial image sequence after face alignment. Considering the redundancy between adjacent frames in the face video, we keep consistent with the rate of audio features and randomly sample one frame every 960 ms, forming a face image sequence as input to the visual encoder. For each image, the ResNet18 (He et al., 2016) pre-trained on the AffectNet emotion dataset (Mollahosseini et al., 2017) is adopted as backbone to extract a 512-dimensional spatial feature vector. Correspondingly, for a face video, the visual feature sequence $I_{v}\in {\mathbb{R}}^{N_{t}\times d_{v}}$ is obtained, with *d*_*v*_ = 512.

#### 3.1.4. Semantic enhancement module in audio/visual encoder

**Figure 2:**
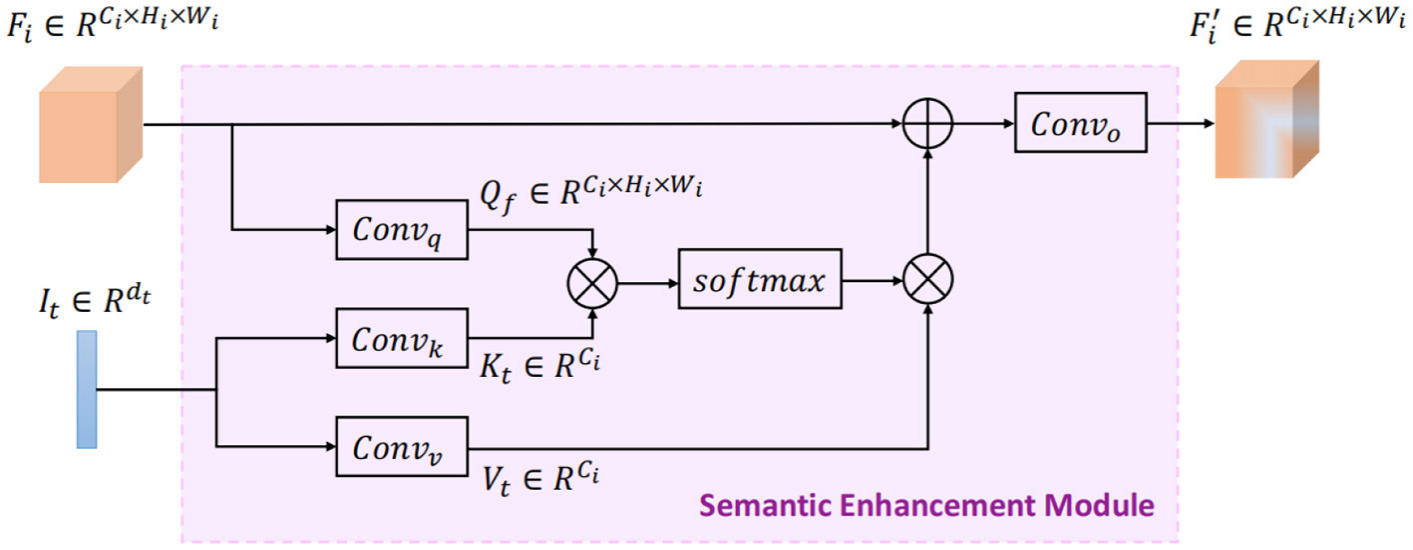
The semantic enhancement module (SEM) in audio/visual encoder.

In order to guide the audio and visual representation learning, a semantic enhancement module (SEM) is designed to infuse high-level semantic information during audio and visual feature encoding. The implementation of SEM is based on the cross-attention mechanism. As shown in Figure 2, each SEM takes the feature map $F_{i}\in {\mathbb{R}}^{C_{i}\times H_{i}\times W_{i}}$ from the middle layer of the audio/visual encoder, as well as the semantic features $I_{t}\in {\mathbb{R}}^{d_{t}}$ from the textual encoder as inputs, then outputs the enriched audio/visual feature map $F_{i}^{\prime }\in {\mathbb{R}}^{C_{i}\times H_{i}\times W_{i}}$ with high-level semantic information. Here, *C*_*i*_, *H*_*i*_, and *W*_*i*_ represent the number of channels, the height and width of the feature map after the *i*^*th*^ convolution group, respectively.

To retrieve emotion-relevant information from the semantic features to guide audio/visual representation learning, we use the input audio/visual feature map *F*_*i*_ as query *Q*_*f*_ and the input semantic features *I*_*t*_ as key *K*_*t*_ and value *V*_*t*_ during the cross-attention computation, implying a latent adaption from text to audio/visual modality. Formally, the query, key, and value are computed as follows:


(1) $$\begin{array}{l} Q_{f}=Conv_{q}(F_{i})\in {\mathbb{R}}^{C_{i}\times H_{i}\times W_{i}};\;K_{t}=Conv_{k}(I_{t})\in {\mathbb{R}}^{C_{i}};\\ V_{t}=Conv_{v}(I_{t})\in {\mathbb{R}}^{C_{i}} \end{array}$$

where *Conv*_*q*_, *Conv*_*k*_, and *Conv*_*v*_ are projection functions with 1 × 1 convolution operation. Next we compute the dot products of *Q*_*f*_ with *K*_*t*_, divided by $\sqrt{C_{i}}$, and then apply a softmax operator to obtain the weights on *V*_*t*_. Note that *Q*_*f*_ is first flattened to unroll the spatial dimensions of feature map for proper calculation, yielding $Q_{f}^{\prime }\in {\mathbb{R}}^{C_{i}\times H_{i}W_{i}}$. The output matrix is formulated as:


(2) $$\begin{array}{l} E_{att}=\text{softmax}\left(\frac{{Q_{f}^{\prime }}^{T}K_{t}}{\sqrt{C}}\right){V_{t}}^{T}\in {\mathbb{R}}^{H_{i}W_{i}\times C_{i}} \end{array}$$

Then, the attention map *E*_*att*_ is reshaped to the same size of the input audio/visual feature map through the unflatten and transpose operations, yielding $E_{att}^{\prime }\in {\mathbb{R}}^{C_{i}\times H_{i}\times W_{i}}$. Finally, the enriched feature map $F_{i}^{\prime }$ is output with semantic guided information as follows:


(3) $$\begin{array}{l} F_{i}^{\prime }=\text{ReLU}\left(Conv_{o}\left(F_{i}+\text{LN}\left(E_{att}^{\prime }\right)\right)\right)\in {\mathbb{R}}^{C_{i}\times H_{i}\times W_{i}} \end{array}$$

where *Conv*_*o*_ denotes 1 × 1 convolution operation, LN represents layer normalization (Ba et al., 2016), and ReLU is the nonlinear activation function.

Conventionally, the audio encoder backbone VGGish contains four convolution groups, and the visual backbone ResNet18 contains five convolution groups, as shown in Figure 1. We empirically insert the semantic enhancement module after the second and last convolution group (conv2 and conv4) of VGGish, and the third and last convolution group (conv3_x and conv5_x) of ResNet18, respectively. The effect of the numbers of SEM in audio/visual encoder will be discussed in the Section 4.

Equipped with SEM, the feature sequences output from the audio and visual encoders are enhanced by the high-level semantic information from the text modality, denoted as $I_{a}^{t}$ and $I_{v}^{t}$, respectively.

### 3.2. Cross-modal encoder

After obtaining the semantically enhanced audio and visual feature sequences through the above-mentioned unimodal encoders, a cross-modal encoder is required to model the cross-modality relationship between audio and visual modalities. The classical approach is to apply the pair-wise bidirectional cross-attention (Tsai et al., 2019). In the case of considering two modalities (audio and visual), this approach needs four cross-modal Transformer branches, which greatly increases the computational cost. Therefore, we borrow the solution of multimodal bottleneck Transformer (MBT) (Liu et al., 2022) from the field of video understanding, to implement the cross-modal encoder with efficient interactions between audio and visual feature sequences.

**Figure 3:**
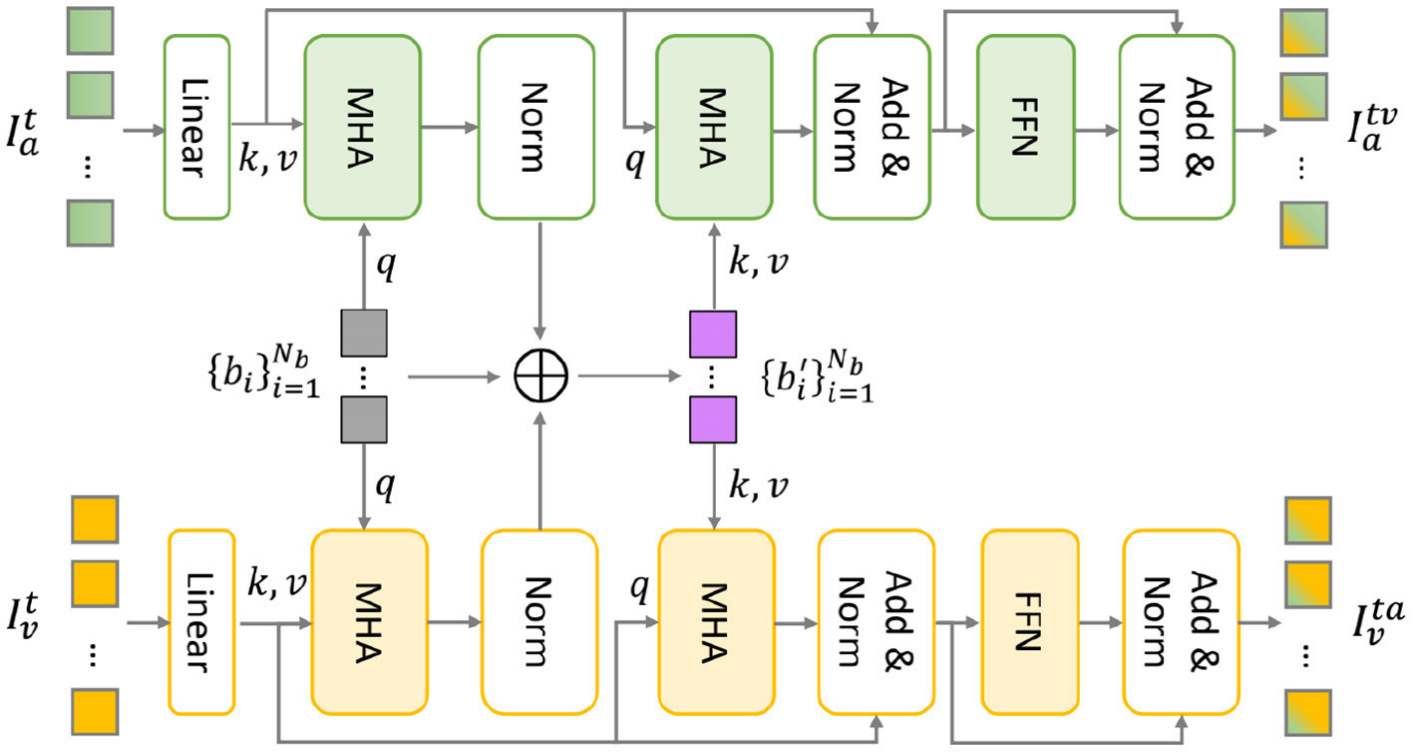
The multimodal bottleneck transformer (MBT) architecture (Liu et al., 2022).

As shown in Figure 3, the MBT architecture contains two parallel Transformer branches, serving audio and visual feature sequences for temporal modeling, respectively. The attention bottlenecks are used as the information bridge to exchange complementary information and remove redundancy between audio and visual modalities. Accordingly, the audio and visual feature sequences are mutually reinforced through audio-visual temporal interaction.

Specifically, linear projection is first performed to map the audio/visual features into the identical dimension *d*_*m*_. Then, a set of bottleneck tokens ${\left\{b_{i}\right\}}_{i=1}^{N_{b}}$ are introduced to aggregate audiovisual temporal information. Following Liu et al. (2022), we use the same two-stage cross-modal interaction through feature compression and expansion.

The first interaction stage implies a process of feature compression using a multi-head attention (MHA) layer in the audio and visual Transformer branch, respectively. By treating bottleneck tokens as *query* and audio/visual tokens as *key*−*value* pairs, the emotional-relevant multimodal information is condensed into the corresponding audio/visual/bottleneck tokens. Through summing up these three tokens, the multimodal information is aggregated into ${\left\{b_{i}^{\prime }\right\}}_{i=1}^{N_{b}}$.

Subsequently, the second interaction stage is propagating the aggregated multimodal emotional information to the target audio/visual modality through another multi-head attention layer in the audio and visual Transformer branch, respectively. Different from feature compression, the bottleneck tokens are treated as *key*−*value* and audio/visual tokens as *query* during this process of feature expansion. Through this two-stage cross-modal attention, audio and visual representations are reinforced with complementary information through interaction with another modality and different time stamps.

Next, the audio and visual features are separately fed into a feed-forward network (FFN) layer to further increase non-linearity, resulting in the reinforced audio and visual feature sequences, denoted as $I_{a}^{tv}$ and $I_{v}^{ta}$, respectively.

### 3.3. Attentive decision fusion

Finally, the mutually enhanced audio and visual feature sequences are separately input into a global average pooling (GAP) layer and an MLP to obtain unimodal decisions $S_{x}\in {\mathbb{R}}^{M}$, where *M* represents the number of emotion categories and *x*∈{*a, v*} represents the audio or visual modality. Meanwhile, the semantic feature vector *I*_*t*_ is input into another MLP to get the textual decision $S_{t}\in {\mathbb{R}}^{M}$.

When fusing these unimodal emotion decisions, we perform attention-based decision fusion to assign higher weights to emotionally salient modality. The unimodal decisions are first concatenated as $S_{con}=\left[S_{a};S_{v};S_{t}\right]\in {\mathbb{R}}^{M\times 3}$. Then, the attention weights are calculated as:


(4) $$\begin{array}{l} S^{\prime }=\text{tanh}\left(W_{1}S_{con}\right) \end{array}$$

(5) $$\begin{array}{l} \alpha_{att}=\text{softmax}\left(W_{2}^{T}S^{\prime }\right) \end{array}$$

where $W_{1}\in {\mathbb{R}}^{M\times M}$ and $W_{2}\in {\mathbb{R}}^{M\times 3}$ are both trainable parameters, and the attention weight $\alpha_{att}\in {\mathbb{R}}^{1\times 3}$. Finally, the emotion prediction is output after attentive weighted fusion:


(6) $$\begin{array}{l} \text{output}=S_{con}\alpha_{att}^{T} \end{array}$$

## 4. Experiments

### 4.1. Datasets

To validate the effectiveness of our proposed method, we conduct experiments on two popular video emotion recognition benchmarks, including the Interactive Emotional Dyadic Motion Capture dataset (IEMOCAP) (Busso et al., 2008) and the CMU Multimodal Opinion Sentiment and Emotion Intensity dataset (CMU-MOSEI) (Zadeh and Pu, 2018):

- IEMOCAP consists of 10 performers, five males and five females, who conduct dialogues in pairs to record 151 videos. These videos are segmented into 10,039 utterances and annotated at the utterance level. Six categorical emotions are considered in this work, namely happiness, sadness, angry, frustrated, excited and neutral.
- CMU-MOSEI contains 3,228 video monologs of 1,000 speakers collected from the YouTube website. Annotation of discrete emotion is performed on 23,453 video clips with a total of six emotion categories: anger, disgust, fear, happiness, sadness, and surprise.

For a fair comparison, we use the raw data reorganized by Dai et al. (2021) to implement fully end-to-end training. Specifically, the train/valid/test set of IEMOCAP includes 5,162, 737, and 1,481 samples, respectively, and the train/valid/test split of CMU-MOSEI dataset corresponds to 14,524, 1,765, and 4,188 video clips, respectively. Note that both datasets are multi-labeled at the utterance level and the statistics are shown in Table 1.

**Table 1:** Statistics of the IEMOCAP and CMU-MOSEI datasets used in this work.

	**IEMOCAP**					
	**>Happiness**	**>Anger**	**>Excited**	**>Frustrated**	**>Sadness**	**>Neutral**
Train	398	757	736	1,298	759	1,214
Valid	62	112	92	180	118	173
Test	135	234	213	371	207	321
	**CMU-MOSEI**					
	**Happiness**	**Anger**	**Disgust**	**Surprise**	**Sadness**	**Fear**
Train	7,587	3,267	2,738	1,465	4,026	1,263
Valid	945	318	273	197	509	169
Test	2,220	1,015	744	393	1,066	371

### 4.2. Evaluation metrics

We use the same metrics adopted in Dai et al. (2021): the average binary accuracy (Avg. Acc) and the average *F*_1_ (Avg. *F*_1_) for IEMOCAP, and the average binary weighted accuracy (Avg. WA) and the average *F*_1_ for CMU-MOSEI. These metrics can be formulated as follows:


(7) $$\begin{array}{c} \text{Avg. Acc}=\frac{1}{C}\overset{C}{\underset{i=1}{\sum }}{\text{Acc}}_{i} \end{array}$$

(8) $$\begin{array}{c} \text{Avg. WA}=\frac{1}{C}\overset{C}{\underset{i=1}{\sum }}{\text{WA}}_{i} \end{array}$$

(9) $$\begin{array}{c} \text{Avg.}F_{1}=\frac{1}{C}\overset{C}{\underset{i=1}{\sum }}{F_{1}}_{i} \end{array}$$

where *C* is the number of emotion categories, Acc_*i*_, WA_*i*_, and _*F*_1_*i*_ denotes the binary accuracy, binary weighted accuracy and *F*_1_ score of the *i*^*th*^ emotion category, respectively:


(10) $$\begin{array}{c} {\text{Acc}}_{i}=\frac{TP}{P+N} \end{array}$$

(11) $$\begin{array}{c} {\text{WA}}_{i}=\frac{TP\times N/P+TN}{2N} \end{array}$$

(12) $$\begin{array}{c} {F_{1}}_{i}=\frac{2TP}{2TP+FP+FN} \end{array}$$

In which *P* and *N* denote the total number of positive and negative samples, respectively, *TP*/*TN* denotes the number of positive/negative samples that are correctly predicted, *FP*/*FN* is the number of negative/positive samples that are incorrectly predicted.

Considering the unbalanced distribution of emotion categories, the Avg. *F*_1_ is used as the main evaluation indicator during the training process.

### 4.3. Implementation details

**Data preprocessing:** For the input audio, log mel-spectrogram is first calculated by using 64 mel-spaced frequency bins on the spectrum obtained from a short-time Fourier transform applying 25 ms windows every 10 ms. The log mel-spectrogram is divided into non-overlapping 960 ms segments that form the input to the audio encoder. The OpenFace (Baltrusaitis et al., 2018) toolkit is utilized to perform face detection and alignment from original videos. After obtaining the facial image sequence from OpenFace, we consider the redundancy between adjacent frames and randomly sample one frame within every 960ms-long duration for each video, yielding the input to the visual encoder. In addition, this sampling operation enables audio and visual features to be temporally aligned at the video level.

**Network parameters:** For the audio encoder backbone VGGish, the output feature dimension is *d*_*a*_ = 128. The output feature dimension of visual encoder backbone ResNet18 is *d*_*v*_ = 512. The pre-trained BERT (*bert-base-uncased*) provided in the HuggingFace library (Wolf et al., 2020) is used as textual encoder. The base BERT model contains 12 layers with a hidden dimension of 768, therefore the semantic feature *I*_*t*_ (i.e., the class token “CLS” of the output layer) is a 768-dimensional vector. For cross-modal encoder, the number of bottleneck tokens of MBT is insensitive and set to *N*_*b*_ = 4 according to the conclusions in Liu et al. (2022), the number of attention heads in multi-head attention layers is 8, the hidden dimension is *d*_*m*_ = 64 and sine-cosine positional encoding is used to preserve the temporal information in the audio/visual feature sequence. The number of floating point operations per second (FLOPs) is 7.22 × 10^9^, the number of parameters is 173M, and the recognition time of one video is around 0.2 s.

**Training parameters:** Regarding the loss function, since both IEMOCAP and CMU-MOSEI datasets are multi-labeled, video emotion recognition is regarded as a multi-label binary classification task in this work, and the binary cross-entropy loss is adopted and weighted by the ratio of the number of positive and negative samples to alleviate the problem of unbalanced sample distribution. Adam optimizer is adopted with a mini-batch size of 8 and the initial learning rate is 1e-4 with early-stopping to prevent overfitting. For the audio and visual encoder backbones, we freeze the first two convolution groups of VGGish and the first three convolution groups of ResNet18, and use a smaller learning rate 1e-5 to fine-tune the rest parameters. The whole framework is implemented using PyTorch on one NVIDIA TITAN RTX GPU.

### 4.4. Results and analysis

#### 4.4.1. Comparison with the state-of-the-art

We compare our model with the following state of the art (SOTA) works where the audio, visual and text modalities are considered: (1) Late Fusion LSTM (LF-LSTM), where each modality uses an individual LSTM to extract global features followed by an MLP for unimodal decision, and the final prediction is obtained by weighted fusion; (2) Late Fusion Transformer (LF-TRANS) which is similar to LF-LSTM except that the Transformer models are used instead of LSTMs to model the temporal dependency for each modality; (3) EmoEmbs (Dai et al., 2020) where three LSTMs are adopted to obtain the global features for each modality and generates modality-specific emotion embeddings through mapping the GloVe textual emotion embeddings to the non-textual modalities respectively, and finally the similarity scores between the emotion embedding and the global features are calculated and fused to get the final prediction; (4) MulT (Tsai et al., 2019) that employs six cross-modal attention modules for any two pairs of the three modalities, and then three self-attention modules to collect temporal information within each modality. Finally the concatenated features are passed through the fully-connected layers to make predictions; (5) BIMHA (Wu et al., 2022) mainly consists of two parts: inter-modal interaction and inter-bimodal interaction, where the outer product is first used to represent three pairs of bimodal global features and then the bimodal attention is calculated *via* an extended multi-head attention mechanism; (6) CMHA (Zheng et al., 2022) where the core is connecting multiple multi-head attention modules in series, to model the interactions between two unimodal feature sequences first and then with the third one. Additionally, the sequential order of modality fusion is considered, resulting in three similar fusion modules but in different orders of fusion; (7) FE2E (Dai et al., 2021) which is a fully end-to-end framework, where the textual features are extracted from a pre-trained ALBERT model and the audio and visual features are extracted from two pre-trained CNNs, each followed by a Transformer to encode the sequential representations, and then three MLPs are adopted to make unimodal decision and weighted fusion is performed to output predictions; (8) MESM (Dai et al., 2021) which is similar to FE2E, except that the original CNN layers are replaced with cross-modal sparse CNN blocks to reduce the computational overhead.

**Figure 4:**
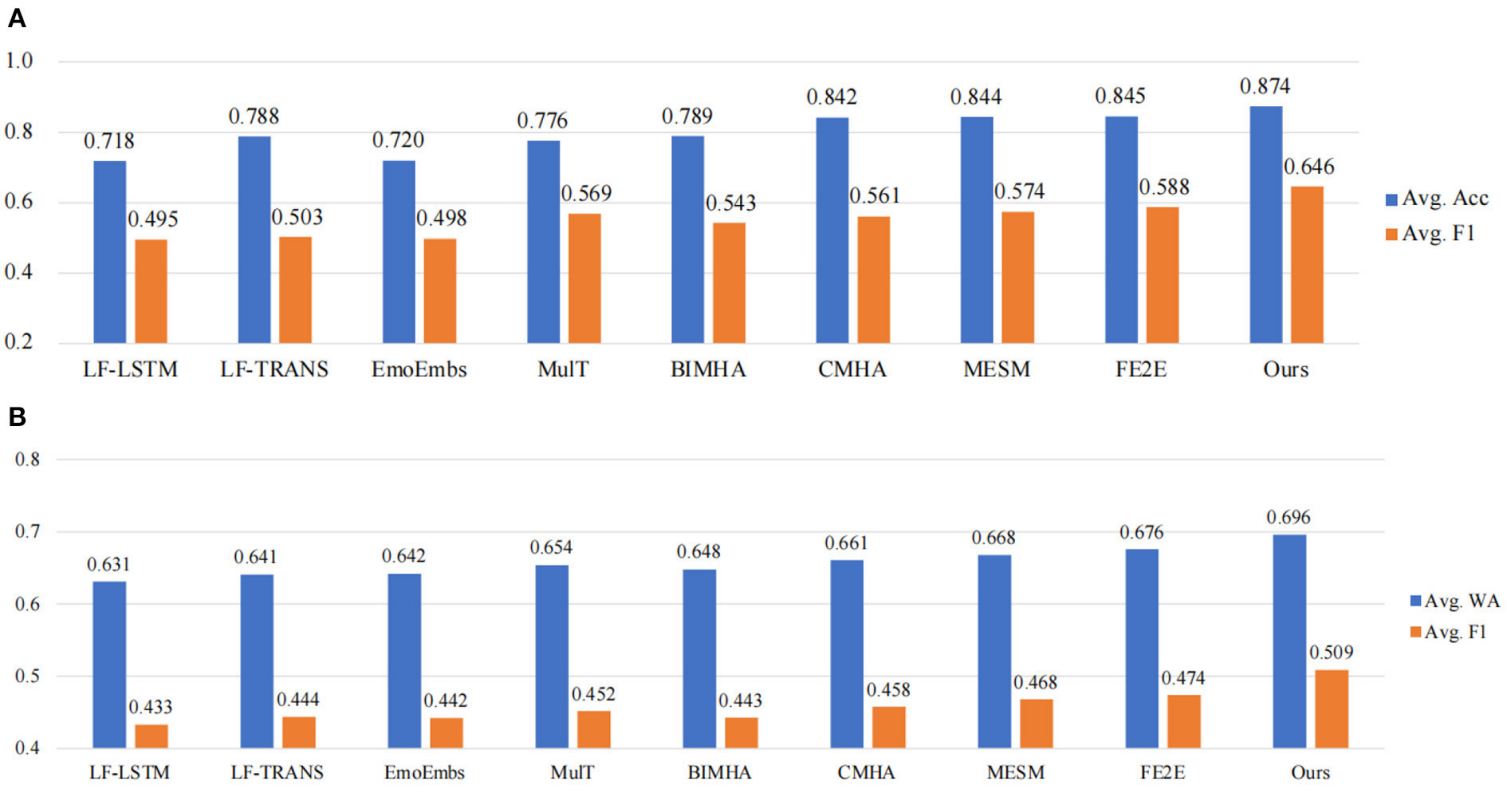
Comparison with state-of-the-art methods on two benchmark datasets. **(A)** Comparison results on IEMOCAP. **(B)** Comparison results on CMU-MOSEI.

The results are shown in Figure 4. Note that all the SOTA results are based on tri-modal decisions from audio, visual and text. It should also be mentioned that, the first five methods (LF-LSTM, LF-TRANS, EmoEmbs, MulT, and BIMHA) are based on hand-crafted features, where 142-dimensional audio features are extracted using the DisVoice toolkit (Vasquez-Correa et al., 2019), 35-dimensional visual features are extracted *via* the OpenFace toolkit (Baltrusaitis et al., 2018), and 300-dimensional word embeddings are extracted using the pre-trained GloVe Pennington et al. (2014). To evaluate the significance of our experimental results, following (Zhang et al., 2021a), the paired *t*-test is performed with a default significance level of 0.05. As it can be seen, our proposed model outperforms all the SOTA works on both IEMOCAP and CMU-MOSEI datasets. The average accuracy reaches 0.874 and the average *F*_1_ is 0.646 on IEMOCAP dataset. On CMU-MOSEI dataset, our model also achieves the highest average weighted accuracy of 0.696 and an average *F*_1_ of 0.509. In addition, the end-to-end methods achieve superior recognition results compared to the two-stage methods based on hand-crafted features, indicating that joint optimization of unimodal feature extraction and multimodal fusion helps improve the performance of video emotion recognition. It should also be mentioned that MESM (Dai et al., 2021) was equipped with cross-modal attention in the feature encoding stage with the aim to make CNN encoders sparse, however, modeling the emotion dependency between audio-video sequences, as a key for multimodal emotional representation learning, was neglected in their whole framework. Compared with MESM, our proposed MER-SEM-MBT obtains better performance due to additional audio-visual temporal interaction.

We also list the binary classification results regarding each emotion category to make a deeper comparison. The detailed results are listed in Table 2, and the best results are bolded. One can notice that our proposed MER-SEM-MBT model achieves the best results on majority emotion category. In addition, we verify a variation of the proposed model by removing the textual decision and the corresponding results are listed in the last row. Under this circumstance, our proposed method, equipped with SEM and MBT modules, still obtains a comparative performance without a textual decision.

**Table 2:** Binary classification results of each emotion category on IEMOCAP and CMU-MOSEI datasets.

	**IEMOCAP**											
**Models**	**Happiness**		**Anger**		**Sadness**		**Excited**		**Frustrated**		**Neutral**	
	**Acc**	*F*_1_	**Acc**	*F*_1_	**Acc**	*F*_1_	**Acc**	*F*_1_	**Acc**	*F*_1_	**Acc**	*F*_1_
LF-LSTM^†^	0.672	0.376	0.712	0.494	0.782	0.540	0.793	0.572	0.682	0.515	0.665	0.470
LF-TRANS^†^	0.852	0.376	0.819	0.507	0.874	0.574	0.853	0.573	0.605	0.493	0.724	0.497
EmoEmbs (Dai et al., 2020)^†^	0.696	0.383	0.659	0.489	0.808	0.530	0.735	0.583	0.685	0.520	0.736	0.487
MulT (Tsai et al., 2019)^†^	0.800	0.468	0.779	0.607	0.835	0.654	0.769	0.580	0.724	0.570	0.749	0.537
BIMHA (Wu et al., 2022)^††^	0.834	0.432	0.772	0.576	0.838	0.637	0.783	0.561	0.739	0.542	0.764	0.509
CMHA (Zheng et al., 2022)^††^	0.890	0.458	0.886	0.611	0.883	0.616	0.879	0.605	0.751	0.563	0.765	0.512
MESM (Dai et al., 2021)^†^	0.895	0.473	0.882	0.628	0.886	0.622	0.883	0.612	0.749	0.584	0.770	0.520
FE2E (Dai et al., 2021)^†^	**0.900**	0.448	0.887	0.639	0.891	0.657	0.891	0.619	0.712	0.578	0.791	0.584
**MER-SEM-MBT** (Our full model)	0.891	**0.577**	**0.894**	**0.665**	**0.924**	**0.721**	**0.905**	**0.677**	**0.797**	**0.613**	**0.832**	**0.623**
**MER-SEM-MBT** (Ours w/o textual decision)	0.889	0.546	0.893	0.662	0.918	0.701	0.892	0.643	0.794	0.602	0.827	0.613
	**CMU-MOSEI**											
**Models**	**Happiness**		**Sadness**		**Anger**		**Surprise**		**Fear**		**Disgust**	
	**WA**	*F*_1_	**WA**	*F*_1_	**WA**	*F*_1_	**WA**	*F*_1_	**WA**	*F*_1_	**WA**	*F*_1_
LF-LSTM^†^	0.613	0.732	0.634	0.472	0.645	0.471	0.571	0.206	0.617	0.222	0.705	0.498
LF-TRANS^†^	0.606	0.729	0.601	0.455	0.653	0.477	0.621	0.242	0.621	0.240	0.744	0.519
EmoEmbs (Dai et al., 2020)^†^	0.612	0.719	0.605	0.475	0.668	0.494	0.633	0.240	0.638	0.234	0.696	0.487
MulT (Tsai et al., 2019)^†^	0.672	**0.754**	0.640	0.483	0.649	0.475	0.614	0.256	0.629	0.253	0.716	0.493
BIMHA (Wu et al., 2022)^††^	0.658	0.721	0.626	0.479	0.653	0.474	0.625	0.249	0.618	0.247	0.705	0.489
CMHA (Zheng et al., 2022)^††^	0.652	0.721	0.642	0.467	0.659	0.491	0.645	0.266	0.634	0.273	0.736	0.532
MESM (Dai et al., 2021)^†^	0.641	0.723	0.630	0.466	0.668	0.493	0.657	0.272	0.658	0.289	0.756	0.564
FE2E (Dai et al., 2021)^†^	0.654	0.726	0.652	0.490	0.670	**0.496**	0.667	0.291	0.638	0.268	0.777	0.571
**MER-SEM-MBT** (Our full model)	**0.673**	0.753	**0.668**	**0.538**	**0.687**	0.495	0.676	**0.330**	**0.672**	**0.319**	**0.802**	**0.616**
**MER-SEM-MBT** (Ours w/o textual decision)	0.672	0.749	0.655	0.531	0.673	0.491	0.660	0.328	0.659	0.312	0.787	0.612

*P* < 0.05 for paired *t*-test.

† denotes the results are from Dai et al. (2021), and

†† means our reproduction using the same data split as other experiments. The bold values are indicated to highlight the best results.

#### 4.4.2. Ablation study

##### 4.4.2.1. Effect of SEM and MBT

To evaluate the contribution of each design module, we further carry out experiments on different model variants by ablating either SEM or MBT, corresponding to MER-MBT (without SEM in unimodal audio/visual encoder) and MER-SEM (without MBT as the cross-modal encoder) respectively. The results are shown in Table 3, where MER stands for a baseline model with unimodal encoders and late attentive fusion. As we can see, either MER-SEM or MER-MBT yields a sub-optimal performance on both IEMOCAP and CMU-MOSEI datasets. Specifically, when MBT is removed, meaning there is no temporal interactions between audio and visual feature sequences, the modal variant MER-SEM obtains an average *F*_1_ of 0.636 on IEMOCAP dataset with a decrease of 1% compared with our full model MER-SEM-MBT, but still 2.2% better than the baseline MER model benefiting from the semantic guidance from SEM. Similarly, when SEM is removed, the model variant MER-MBT achieves an average *F*_1_ of 0.633 on IEMOCAP, which is 1.3% lower than the full model. Furthermore, if both SEM and MBT modules are removed, i.e., the baseline MER model, the average *F*_1_ only reaches 0.614 on IEMOCAP, which is 3.2% lower than our proposed full model MER-SEM-MBT. This may be due to the fact that the baseline model MER only adopts attentive fusion to aggregate the individual audio and visual decisions without interaction across different modalities. Similar conclusions can also be drawn from the reuslts on the CMU-MOSEI dataset.

**Table 3:** Ablation study results on IEMOCAP and CMU-MOSEI datasets.

**Models**	**SEM**	**MBT**	**LF**	**IEMOCAP**		**CMU-MOSEI**	
				**Avg. Acc**	**Avg. *F*_1_**	**Avg. WA**	**Avg. *F*_1_**
MER	-	-	✓	0.855	0.614	0.682	0.496
MER-SEM	✓	-	✓	0.871	0.636	0.691	0.506
MER-MBT	-	✓	✓	0.868	0.633	0.688	0.504
MER-SEM-MBT	✓	✓	✓	**0.874**	**0.646**	**0.696**	**0.509**

The bold values are indicated to highlight the best results.

##### 4.4.2.2. Effectiveness of SEM in audio/visual encoder

We further analyze the effectiveness of SEM on audio and visual representation learning for audio and visual emotion recognition, respectively. For convenience, we denote the audio emotion recognition as SER and visual emotion recognition as FER. Note that the textual decision is not used in the following experiments. As listed in Table 4, the first/third row represents the SER/FER results from the CNN-Transformer-MLP framework without SEM, where the CNN encoder (VGGish for audio and ResNet18 for video) is for feature extraction from raw data, Transformer is for temporal modeling, and MLP is for classification. The second/fourth row shows the results of SEM being inserted in the unimodal CNN encoder for SER/FER. It can be seen that when SEM is inserted to guide the audio/visual encoder to learn the emotional representation from the semantics, the performances are greatly improved. For SER, the average Acc improves from 0.752 to 0.839 on IEMOCAP dataset with a gain of 8.7% after SEM is used to enhance the representation learning of audio encoder. For FER, the average Acc also achieves a gain of 4.4% in terms of Avg. WA on CMU-MOSEI dataset.

**Table 4:** Unimodal audio/visual emotion recognition results with and without SEM.

**Methods**		**IEMOCAP**		**CMU-MOSEI**	
		**Avg. Acc**	**Avg. *F*_1_**	**Avg. WA**	**Avg. *F*_1_**
SER	w/o SEM	0.752	0.463	0.628	0.424
	w/ SEM	**0.839**	**0.560**	**0.659**	**0.450**
FER	w/o SEM	0.796	0.512	0.631	0.429
	w/ SEM	**0.828**	**0.553**	**0.675**	**0.456**

SER refers to speech emotion recognition, and FER denotes facial expression recognition. All frameworks follow the CNN-Transformer-MLP architecture, the difference is whether SEM is used in the CNN encoder. The bold values are indicated to highlight the best results.

##### 4.4.2.3. Effect of the number of SEMs

**Figure 5:**
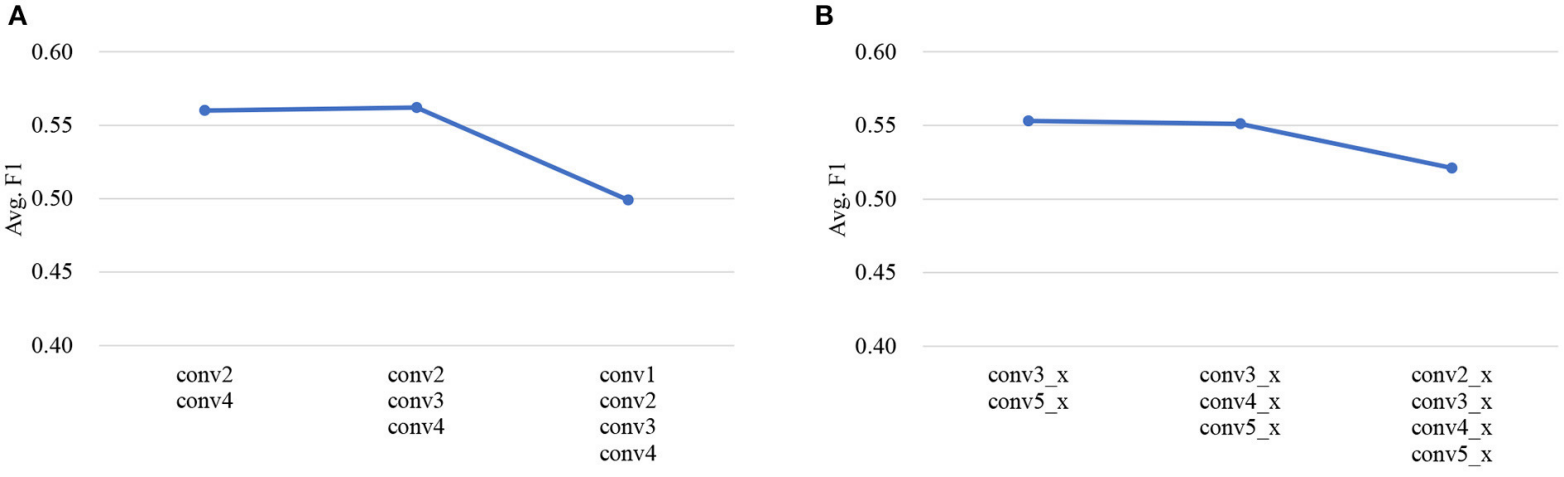
The effect of different numbers of SEMs in audio and visual encoder, respectively. The results are shown in terms of Avg. *F*_1_ on IEMOCAP dataset. **(A)** Audio encoder (VGGish with SEM). **(B)** Visual encoder (ResNet18 with SEM).

As described in Section 3.1.4, SEM is empirically inserted after the second and last (fourth) convolution group for audio encoder backbone VGGish, and the third and last (fifth) convolution group for visual encoder backbone ResNet18, respectively. Here, we conduct experiments on IEMOCAP dataset to explore the effect of different numbers of SEMs in audio/visual encoder, the results are shown in Figure 5. Taking SER for example, the default setting is inserting two SEMs after the second and the fourth convolutional group, respectively. From Figure 5A, we can see that when adding another SEM after the third convolution group of VGGish, the result is close to that of the default setting, and further adding another SEM after the first convolution group results in a significant drop in performance. Similar conclusion can be drawn from Figure 5B for visual encoder. This is probably because the feature maps output from the earlier convolution group mainly contain low-level information, while those from the deeper layers with high-order features are more relevant to emotions, therefore the semantics can better adapt the high-level audio/visual feature maps with emotion-related information.

##### 4.4.2.4. Performance comparison of different cross-modal encoders

To validate the effectiveness of adopting MBT as cross-modal encoder in our proposed framework, we perform audio-visual multi-modal emotion recognition (MER) experiments using different cross-modal encoders. Note that all the methods in this comparative experiment use the same audio and visual encoders, i.e., VGGish for audio and ResNet18 for video (without using semantic information for enhancement), and the same attentive decision fusion as described in Section 3.3. The results are shown in Table 5.

**Table 5:** Audio-visual emotion recognition results using different cross-modal encoders.

**Cross-modal Encoder**	**IEMOCAP**		**CMU-MOSEI**	
	**Avg. Acc**	**Avg. *F*_1_**	**Avg. WA**	**Avg. *F*_1_**
JointAtt (Vaswani et al., 2017)	0.846	0.582	0.667	0.487
Bi-CrossAtt (Tsai et al., 2019)	0.842	0.571	0.671	0.473
MBT (Liu et al., 2022)	**0.859**	**0.592**	**0.676**	**0.491**

The bold values are indicated to highlight the best results.

Concretely, three typical attention-based solutions are compared: (1) joint attention (JointAtt), where the audio and visual feature sequences are temporally concatenated and then input into a vanilla Transformer (Vaswani et al., 2017), therefore the information within these two modalities can be fully communicated; (2) bidirectional cross-attention (Bi-CrossAtt) (Tsai et al., 2019), where two cross-modal Transformer branches are employed, each serves to reinforce a target modality with the features from the other modality *via* learning the attention across the audio and visual feature sequences; (3) multimodal bottleneck attention (MBT) (Liu et al., 2022), which introduces bottleneck tokens as the bridge connecting two Transformer branches, to exchange essential information from one modality to the other through a two-stage cross-modal interaction.

It can be seen that the cross-modal interaction with MBT achieves the highest recognition results on both datasets, indicating that attention bottlenecks, with the advantage of exchanging audio-visual complementary information and reducing redundancy, further enhance the representation learning of audio/visual modalities.

#### 4.4.3. Visualization

**Figure 6:**
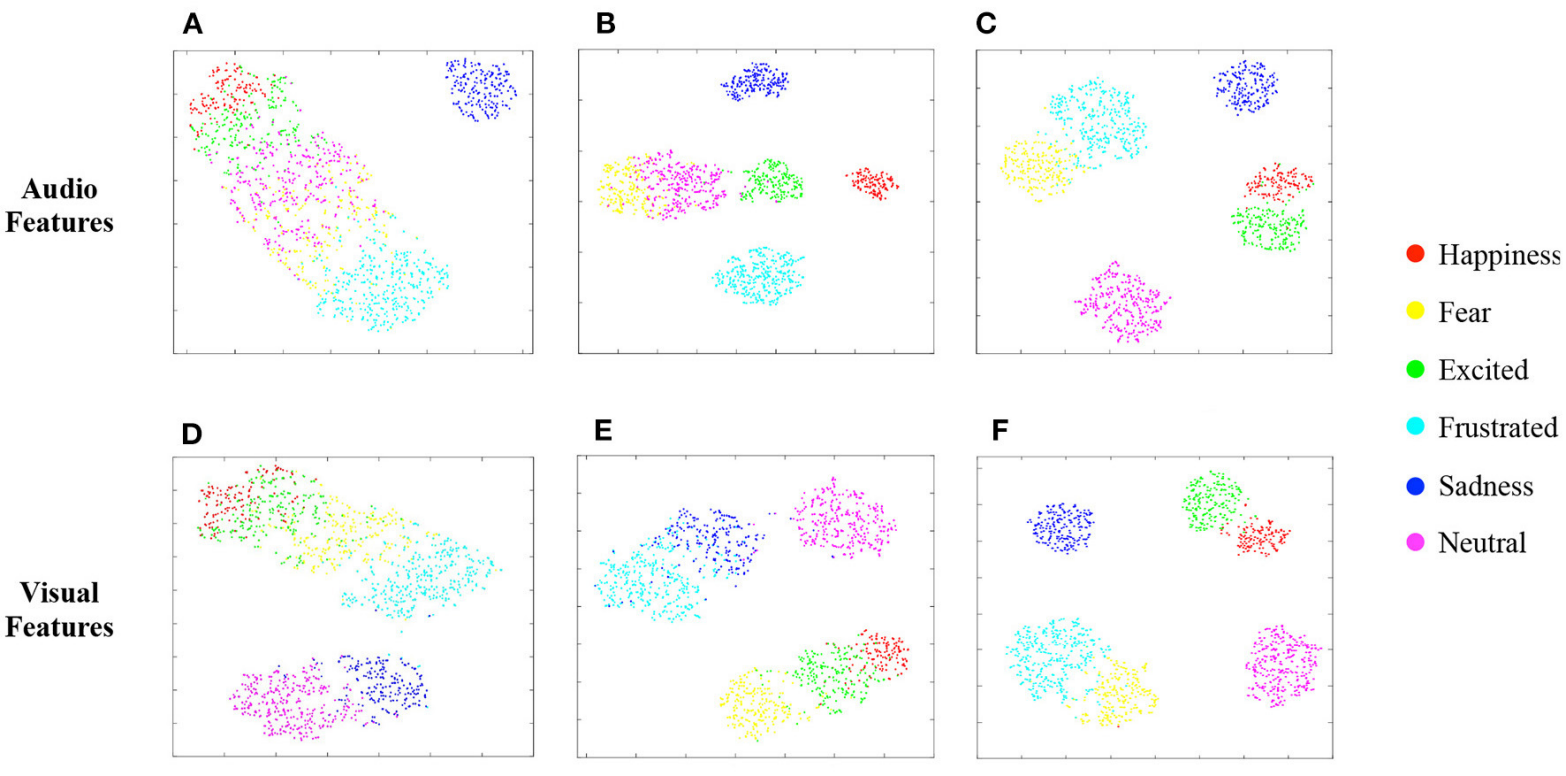
Visualization of audio and visual feature distribution on IEMOCAP. **(A)** SER w/o SEM, w/o MBT. **(B)** SER w/SEM, w/o MBT. **(C)** MER w/SEM, w/MBT. **(D)** FER w/o SEM, w/o MBT. **(E)** FER w/SEM, w/o MBT. **(F)** MER w/SEM, w/o MBT.

We also perform t-SNE (Van der Maaten and Hinton, 2008) to visualize the learned audio and visual features, under three different settings, from the penultimate layer of their MLPs, respectively. Note that the textual decision is not used in the involved models here. Figures 6A, D represents the audio/visual features learned by the unimodal SER/FER model without SEM and MBT, which corresponds to the results in the first/third row of Table 4. As we can see, the learned audio/visual features can not distinguish different emotions well in the absence of additional information from other modalities. When SEM is added in the audio/visual encoder for SER/FER, the enhanced audio/visual features of different emotion categories, as shown in Figures 6B, E, are more discriminatively distributed, which help to improve the emotion recognition performance as compared in Table 4. In addition, when MBT is further added, achieving cross-modal interaction between audio and visual representations, the dually reinforced audio/visual features (corresponds to Figures 6C, F) are more distinguishable, contributing to the best performance.

## 5. Conclusions

In this work, we proposed a multimodal interaction enhanced representation learning method targeting video emotion recognition. The high-level semantic information extracted from the text modality is utilized to enhance audio and visual feature encoding, and the bottleneck Transformer is adopted to further reinforce audio and visual feature sequences through exchanging complementary information while reducing redundancy. Finally, audio, visual, and textual unimodal decisions are fused using attention weights to output the final emotion prediction. Experiments and visualization show that the proposed method achieves state-of-the-art video emotion recognition results. In the future, we are interested to leverage self-supervised learning methods to learn better emotional-salient representations by exploring the correlations among audio, visual, and text modalities.

## Data availability statement

Publicly available datasets were analyzed in this study. This data can be found at: https://sail.usc.edu/iemocap/ and http://multicomp.cs.cmu.edu/resources/cmu-mosei-dataset/.

## Author contributions

XX, YZ, and DJ contributed to conception and design of the study. XX wrote the first draft of the manuscript. YZ and DJ revised the manuscript. All authors contributed to manuscript revision, read, and approved the submitted version.
